# The Selective Loss of Purkinje Cells Induces Specific Peripheral Immune Alterations

**DOI:** 10.3389/fncel.2021.773696

**Published:** 2021-11-30

**Authors:** Carlos del Pilar, Rafael Lebrón-Galán, Ester Pérez-Martín, Laura Pérez-Revuelta, Carmelo Antonio Ávila-Zarza, José Ramón Alonso, Diego Clemente, Eduardo Weruaga, David Díaz

**Affiliations:** ^1^INCyL, Institute for Neuroscience of Castile and Leon, Universidad de Salamanca, Salamanca, Spain; ^2^IBSAL, Institute of Biomedical Research of Salamanca, Salamanca, Spain; ^3^Grupo de Neuroinmuno-Reparación, Hospital Nacional de Parapléjicos, Toledo, Spain; ^4^SESCAM (Servicio de Salud de Castile-La-Mancha), Castilla–La Mancha, Spain; ^5^Applied Statistics Group, Department of Statistics, Universidad de Salamanca, Salamanca, Spain; ^6^Instituto de Alta Investigación, Universidad de Tarapacá, Arica, Chile

**Keywords:** selective neurodegeneration, neuroinflammation, brain infiltration, biomarkers, peripheral immune alterations

## Abstract

The progression of neurodegenerative diseases is reciprocally associated with impairments in peripheral immune responses. We investigated different contexts of selective neurodegeneration to identify specific alterations of peripheral immune cells and, at the same time, discover potential biomarkers associated to this pathological condition. Consequently, a model of human cerebellar degeneration and ataxia -the Purkinje Cell Degeneration (PCD) mouse- has been employed, as it allows the study of different processes of selective neuronal death in the same animal, i.e., Purkinje cells in the cerebellum and mitral cells in the olfactory bulb. Infiltrated leukocytes were studied in both brain areas and compared with those from other standardized neuroinflammatory models obtained by administering either gamma radiation or lipopolysaccharide. Moreover, both myeloid and lymphoid splenic populations were analyzed by flow cytometry, focusing on markers of functional maturity and antigen presentation. The severity and type of neural damage and inflammation affected immune cell infiltration. Leukocytes were more numerous in the cerebellum of PCD mice, being located predominantly within those cerebellar layers mostly affected by neurodegeneration, in a completely different manner than the typical models of induced neuroinflammation. Furthermore, the milder degeneration of the olfactory bulb did not foster leukocyte attraction. Concerning the splenic analysis, in PCD mice we found: (1) a decreased percentage of several myeloid cell subsets, and (2) a reduced mean fluorescence intensity in those myeloid markers related to both antigen presentation and functional maturity. In conclusion, the selective degeneration of Purkinje cells triggers a specific effect on peripheral immune cells, fostering both attraction and functional changes. This fact endorses the employment of peripheral immune cell populations as concrete biomarkers for monitoring different neuronal death processes.

## Introduction

Neurodegenerative diseases are generally accompanied by local inflammatory reactions that have been associated with an altered immune cell infiltration into the damaged nervous system (Zlokovic, 2011; Ransohoff and Brown, 2012). This phenomenon is critical for microglial regulation, a pivotal player in neuroinflammation known to be involved in the progression of the neurodegenerative process (Boyko et al., 2017; McManus and Heneka, 2017). Interestingly, alterations have not only been observed in the central nervous system, but also at the peripheral level, multiple sclerosis being a good example in which a clear participation of immune cells has been reported (Fakhoury, 2016). Moreover, changes in the peripheral immune system have also been described in Alzheimer’s disease (AD; Ciaramella et al., 2016; Gupta et al., 2018; Le Page et al., 2018), Huntington’s disease (Björkqvist et al., 2008), Parkinson’s disease (PD; Scherzer et al., 2007; Fuzzati-Armentero et al., 2019) and amyotrophic lateral sclerosis (ALS; Liu and Wang, 2017; Zhao et al., 2017). These alterations include changes in the distribution and activation of lymphocytes and macrophages (Lucin and Wyss-Coray, 2009), the latter usually presenting a pro-inflammatory phenotype in humans suffering from neurodegenerative disorders (González and Pacheco, 2014). Furthermore, increased levels of pro-inflammatory cytokines have been detected in both blood and cerebrospinal fluid of patients with AD or PD (Boyko et al., 2017; Chen et al., 2018). Therefore, a better comprehension of peripheral immune responses is becoming essential to understand neurodegenerative diseases to predict, halt or delay their progression.

All the studies mentioned above have addressed neurodegenerative diseases in which distinct neural types perish and, ultimately, variable brain regions result affected. In this study, we wanted to ascertain how peripheral immune cells are influenced by selective neurodegeneration, i.e., by the death of a specific neuronal population. Our aim was to elucidate the behavior of such immune cells in this pathological condition and uncover potential biomarkers for predicting or monitoring its progression. Here we used the Purkinje Cell Degeneration (PCD) mouse, an excellent model of human cerebellar degeneration and ataxia (Shashi et al., 2018; Karakaya et al., 2019; Sheffer et al., 2019). Humans affected by this impairment (as well as PCD mice) hold a mutation in the *CCP1* gene (also known as *AGTPBP1* or *NNA1*) that leads to the postnatal loss of the Purkinje cells in the cerebellum (Mullen et al., 1976; Wang and Morgan, 2007), both species displaying very similar pathological alterations (Shashi et al., 2018; Karakaya et al., 2019; Sheffer et al., 2019). Interestingly, PCD mice also experience the loss of mitral cells in the olfactory bulb (OB). Both neurodegenerative events occur at different and well-defined periods of time (Wang and Morgan, 2007). More precisely, Purkinje cell loss starts at postnatal day 18 (P18) and progresses quickly in such a way that at P25 about 50% of Purkinje cells in the vermis have degenerated, at P30 these neurons mainly survive in the nodulus (lobule X) and ventral side of the uvula (lobule IX), and at P40 only a few Purkinje cells persist, most of which are in lobule X (Mullen et al., 1976; Wang and Morgan, 2007). Moreover, mitral cell degeneration in the OB begins around P60 and then advances more slowly for an additional 2 months (Greer and Shepherd, 1982; Valero et al., 2006). Therefore, the PCD mouse is a suitable model for studying selective neurodegeneration, allowing the assessment of two different and well-characterized scenarios in the same animal and with the advantage of not having to cause neuronal death using invasive techniques that would introduce additional variability.

In particular, we explored possible alterations at the central level by analyzing both leukocyte infiltration and distribution, as well as at the peripheral level by evaluating the phenotype of splenic leukocytes in the PCD mutant mouse in comparison with wild-type (WT) animals. Additionally, in order to compare different neuroinflammatory scenarios, we used lipopolysaccharide (LPS) and gamma radiation to generate two standard models of neuroinflammation in which an enhanced peripheral recruitment into the brain parenchyma has been described (Cazareth et al., 2014; Morganti et al., 2014; Moravan et al., 2016). Our findings highlight the specific effects on peripheral immune cells in the central nervous system caused by the degeneration of Purkinje cells, as well as the intriguing alterations in the myeloid compartment at the peripheral level that could be employed as early biomarkers of this selective neurodegeneration.

## Materials and Methods

### Animals

Both WT and PCD mice from the C57BL/DBA hybrid strain were used. These mice were obtained by mating C57BL/6J and DBA/2J strains, both originally purchased from the Jackson Laboratory (Bar Harbor, ME, United States). Animals were separated into groups depending on their genotype and age at the time of analysis: P15, P20, P25, P30, P40, and P70 (*n* = 5 per age and genotype). These ages were chosen considering the neurodegenerative processes that occur in PCD mice (Mullen et al., 1976; Wang and Morgan, 2007). Additionally, we used another three groups of treated WT mice at P25: one received LPS, and the remaining two groups were gamma-irradiated (*n* = 5 per group). Mice were housed at the animal facility of the University of Salamanca at constant temperature and relative humidity, with a 12/12 h photoperiod, and were fed *ad libitum* with water and special rodent chow (Rodent toxicology diet, B&K Universal G.J., S.L., Barcelona, Spain). Animals were housed, handled, and sacrificed following the guidelines established by European (Directive 2010/63/UE, Recommendation 2007/526/CE) and Spanish (Law 32/2007, RD 53/2013) legislation. All experiments were approved by the Bioethics Committee of the University of Salamanca (reference numbers: #00291 and #00344).

### Genotyping

As PCD mice are not suitable for breeding (Wang and Morgan, 2007), the colony was kept by mating heterozygous animals which are indistinguishable from their WT littermates. As a consequence, the offspring were genotyped by PCR as previously described (Díaz et al., 2012).

### Lipopolysaccharide Administration and Gamma Irradiation

LPS-treated animals were administered with LPS at P24, while irradiated animals were exposed to radiation at P18 or P24. LPS (strain O26:B6, Sigma-Aldrich, St. Louis, MO, United States) was freshly dissolved in saline solution and injected intraperitoneally with a dose of 2 mg/kg body weight, as used in other studies (Cazareth et al., 2014; Fu et al., 2014). In addition, the irradiated animals received a single dose of 3 Gy whole-body gamma irradiation that was supplied using a ^137^Cs source for mice (Gammacell 1000 Elite, 243 cGy/min, 0.662 MeV, MDS Nordion, Ottawa, Canada).

### Tissue Preparation

The mice were deeply anesthetized and subsequently perfused intracardially with 0.9% w/v NaCl for 1 min, followed by Somogyi’s fixative solution containing 4% w/v paraformaldehyde and 15% v/v saturated picric acid in 0.1 M phosphate buffer, pH 7.4 (PB) for 15 min. Brains were dissected out and immersed in the same fixative for 2 h at room temperature. Then, they were rinsed with PB and cryoprotected with 30% w/v sucrose in PB overnight at 4°C. Afterward, cerebella and OBs were cut in 40-μm sagittal or coronal sections, respectively, by employing a freezing-sliding microtome (Jung SM 2000, Leica Microsystems, Wetzlar, Germany). The sections collected were rinsed with PB to remove fixative and sucrose residues and immunostained to visualize the leukocytes.

### Immunofluorescent Labeling

Free-floating sections were washed with phosphate buffered saline, pH 7.4 (PBS; 3 × 10 min) and incubated for 72 h at 4°C under continuous rotary shaking in a medium containing 0.2% v/v Triton X-100, 5% v/v normal donkey serum, and the primary antibodies: rat anti-CD45 (1:1,000; MCA1388; Bio-Rad Laboratories, Hercules, CA, United States), rabbit anti-Iba1 (1:1,000, 019-19741, Wako Pure Chemical Industries, Ltd., Osaka, Japan) or rabbit anti-CD3 (1:200; ab5690, Abcam, Cambridge, United Kingdom) in PBS. These antibodies were used to stain leukocytes (CD45), macrophages/microglia (Iba1) and T lymphocytes (CD3). Only CD45^high^ cells displaying rounded morphology were considered as infiltrated leukocytes (Perego et al., 2016). The CD45 labeling of microglial cells was imperceptible in most cases as previously described (Cuadros et al., 2006) and, when present, it was clearly different in morphology (branched) and intensity (low) compared to the positive staining of leukocytes. Sections were washed with PBS (3 × 10 min) and then incubated in a second medium for 1 h and 30 min at room temperature under continuous rotary shaking. This second medium contained an appropriate secondary antibody conjugated to Cy2 or Cy3 (1:500; Jackson ImmunoResearch Laboratories, Cambridge, United Kingdom) in PBS. Ten minutes prior to the end of the incubation, DAPI (4′,6-diamidino-2-phenylindole; Sigma-Aldrich) at 1:10,000 was added to the medium to obtain a nuclear counterstain. Finally, the sections were rinsed with PBS, mounted on gelatin-coated slides, and covered using a freshly prepared anti-fading mounting medium. Appropriate negative controls without the primary antibodies were performed and no staining was observed in any case.

To better ascertain the peripheral/blood nature of the counted cells and rule out that they were not microglia, we performed an additional experiment consisting of a triple immunohistochemistry against Iba1, CD45 and TMEM119, the latter being expressed on microglia-derived cells but not on recruited blood-derived macrophages (Bennett et al., 2016). Free-floating sections were washed with phosphate buffered saline, pH 7.4 (PBS; 3 × 10 min) and incubated at 4°C for 24 h under continuous rotary shaking in a medium containing 0.5% v/v Triton X-100, 10% v/v normal donkey serum, 0.3 M glycine, and the primary antibodies: rat anti-CD45 (1:1,000; MCA1388; Bio-Rad Laboratories), goat anti-Iba1 (1:1,000; ab5076, Abcam) or rabbit anti-TMEM119 (1:300; ab209064, Abcam) in PBS. Sections were washed with PBS (3 × 10 min) and then incubated in a second medium for 90 min at room temperature under continuous rotary shaking. This second medium contained an appropriate secondary antibody conjugated to Cy2, Cy3, or Cy5 (1:500; Jackson ImmunoResearch Laboratories) in PBS, to mark CD45, TMEM119 or Iba1, respectively. The rest of the protocol was the same as described above.

### Microscopy Visualization and Cell Counting

Sections were observed under either an epifluorescence microscope Olympus Provis AX70 equipped with an Olympus DP70 digital camera (12.5 MP, Olympus, Tokyo, Japan) or a confocal microscope STELLARIS 8 (Leica Microsystems). Epifluorescence images were taken with a spatial resolution of 6,4 px/μm. Confocal images were taken with a spatial resolution of 1.75 px/μm (20X objective), 3.5 px/μm (40X objective) or 14 px/μm (4X magnification with the 40X objective). Digital images were processed using Adobe Photoshop CC 2015 (Adobe Inc., San Jose, CA, United States) to slightly adjust contrast, brightness and color balance.

Leukocytes were manually counted during examination of all sections. For the cerebellar histological analyses, three sections of vermis per animal were chosen, where all cerebellar lobules were clearly seen, and the degeneration occurs earlier (Wang and Morgan, 2007). Regarding the OB, central coronal sections of a one-in-six series (8–10 sections per animal) were analyzed for comparisons, where both mitral cell and glomerular layers were clearly observed, excluding the accessory olfactory bulb (Weruaga et al., 1999). All counts were performed by the same person (CdP) and following the same criteria.

### Analysis of Splenic Subsets by Flow Cytometry

For the exploration of peripheral immune cells, both myeloid and lymphoid populations from four WT and four PCD spleens per age were analyzed by flow cytometry. As soon as the mice were euthanized, their spleens were placed in Hibernate™ medium (Thermo Fisher Scientific, Waltham, MA, United States) until subsequent processing. A single-cell suspension was obtained from spleens by passing them through a 40 μm nylon cell strainer (BD Biosciences, San Jose, CA, United States). Then, the red blood cells were lysed in ACK lysis buffer (0.83% w/v NH_4_Cl, 0.1% w/v KHCO_3_, 1 mM EDTA in distilled H_2_O, pH 7.4; Panreac Química, Barcelona, Spain) and 10^6^ splenocytes were resuspended in 50 μL of staining buffer containing 25 mM HEPES, 2% v/v penicillin/streptomycin, and 10% v/v fetal bovine serum (Cultek, Madrid, Spain) in sterile PBS. Fc receptors were blocked with anti-CD16/CD32 antibodies (10 μg/mL; BD Biosciences) in staining buffer for 10 min at 4°C. Thus, all non-specific bonds between Fc receptors present on immune cells and the subsequent antibodies were interrupted. After blocking, cells were labeled for 30 min at 4°C in darkness using 50 μL of staining buffer containing the corresponding antibodies (see below). Splenocytes were washed twice with staining buffer, recovered by centrifugation at 1,500 rpm for 5 min at room temperature, resuspended in PBS and, finally, analyzed using a FACSCanto™ II cytometer (BD Biosciences).

For the lymphoid subset analysis, we used the following anti-mouse antibodies: FITC-conjugated CD8 (5 μg/mL), PE-conjugated CD4 (2 μg/mL), Pacific Blue-conjugated CD3ε (4 μg/mL; BD Biosciences), PE-Cy5.5-conjugated CD25 (4 μg/mL) and APC-conjugated CD69 (4 μg/mL; Thermo Fisher Scientific). For the myeloid subset analysis, the following anti-mouse antibodies were used: FITC-conjugated Ly-6C (10 μg/mL), PE-conjugated Ly-6G (4 μg/mL), PerCP-Cy5.5-conjugated CD11b (4 μg/mL; BD Biosciences), PE-Cy7-conjugated MHC-II (4 μg/mL), APC-conjugated CD11c (4 μg/mL) and eFluor 450-conjugated F4/80 (4 μg/mL; Thermo Fisher Scientific). The data were analyzed with FACSDiva 6.1 (BD Biosciences) and FlowJo 7.6.4 software (TreeStar Inc., Ashland, OR, United States) from the Flow Cytometry service of the *Hospital Nacional de Parapléjicos*. In all cases, the gating strategy involved the exclusion of dead cells, debris, and doublets.

### Statistical Analysis

Once normality had been checked using the Kolmogorov-Smirnov test, the two-way ANOVA test was employed when the WT and PCD groups at different ages were compared. Once we had confirmed there was no interaction between age and genotype, we performed the Student’s *t*-test in order to evaluate possible differences between PCD and WT mice within each age, or one-way ANOVA and Bonferroni *post hoc* tests to evaluate differences between ages. In addition, the one-way ANOVA test was used to compare several groups at the same age. In this case, Dunnett’s *post hoc* test was performed to compare all groups with their WT or PCD mouse counterparts. The minimum significance level was set at *p* < 0.05, except for detecting possible interactions between factors in the two-way ANOVA test, where significance was considered at *p* < 0.1. All analyses were performed using the IBM SPSS Statistical 25 software (IBM, Armonk, NY, United States).

## Results

### Number of Leukocytes in Wild-Type and Purkinje Cell Degeneration Mouse Olfactory Bulb

Firstly, we investigated the effect of selective mitral cell loss on leukocyte infiltration. For this, we analyzed the number of leukocytes in OB sections from WT and PCD mice at P25, when the degeneration of mitral cells has not started, and P70, when the death of these cells is in progress (Valero et al., 2006). The peripheral nature of the leukocytes counted was verified by ascertaining that they were negatively immunostained for TMEM119, which was only expressed by microglial cells (Supplementary Figure 1).

**FIGURE 1:**
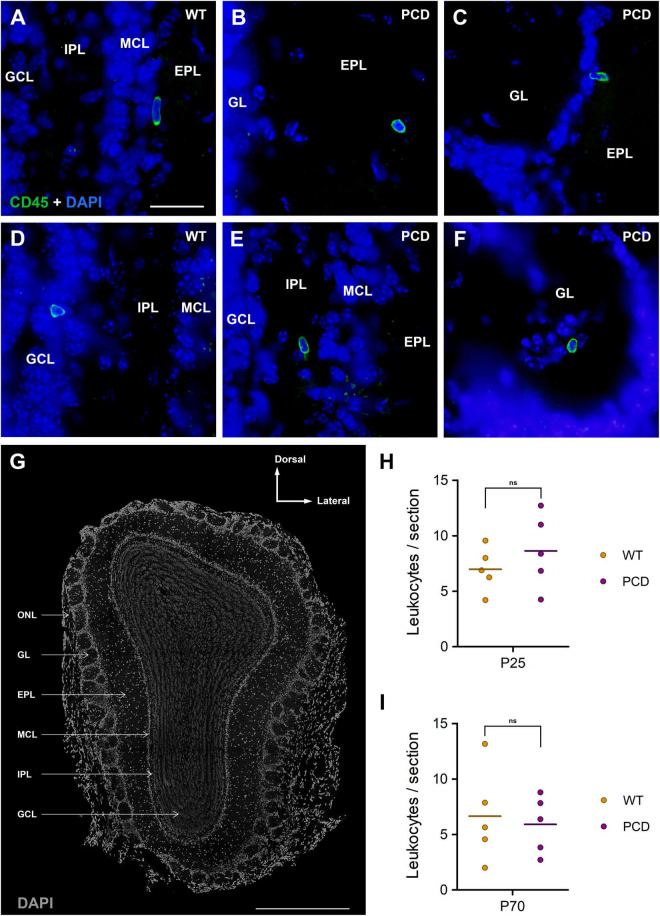
Leukocyte infiltration into the OB of WT and PCD mice. **(A–F)** Immunolabeling for CD45 (green) showing leukocytes in the different layers of the OB of both WT and PCD mice; nuclei are stained with DAPI (blue). **(G)** Typical coronal section of the mouse OB obtained with confocal microscopy; nuclei are stained with DAPI (gray). **(H,I)** Charts showing the quantification of leukocytes per OB section at P25, when the death of mitral cells has not started in PCD mice, and at P70, when the mitral cell loss is in progress. No differences were detected between WT and PCD mice in any case. Data are expressed as dot plots, where each dot represents one individual animal and horizontal lines represent the mean of each group. GL, glomerular layer; EPL, external plexiform layer; MCL, mitral cell layer; IPL, internal plexiform layer; GCL, granule cell layer; ONL, olfactory nerve layer. ns, not significant (*p* > 0.05). Scale bar: 25 μm for **(A–F)**; 500 μm for **(G)**.

Leukocytes (CD45-positive and TMEM119-negative cells) were found in all bulb layers of both WT and PCD mice (Figures 1A–F). The location of the different layers of the OB is depicted in Figure 1G. No statistically significant differences were detected between WT and PCD mice at P25 (*p* = 0.154; Figure 1H) or at P70 (*p* = 0.747; Figure 1I). Therefore, it seems that the moderate affectation and, consequently, the mild neurodegenerative microenvironment in the OB of PCD mice does not trigger leukocyte infiltration.

### Amount and Distribution of Leukocytes in Wild-Type and Purkinje Cell Degeneration Mouse Cerebellum

Afterward, we explored whether the severity of the inflammatory/degenerative process is important for leukocyte infiltration. To this end, we analyzed the number of leukocytes in the cerebellum, where a stronger neurodegenerative and inflammatory microenvironment exits in PCD animals (Baltanás et al., 2013). In this case, we employed WT and PCD mice at P15, P20, P25, P30 and P40, taking into account the temporal progression of the degeneration afflicting PCD mice.

**FIGURE 2:**
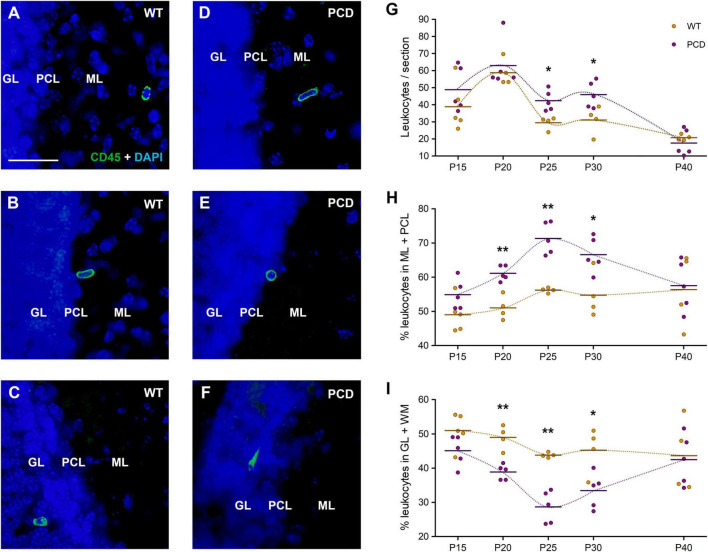
Leukocyte infiltration and distribution in the cerebellum of WT and PCD mice. **(A–F)** Immunolabeling for CD45 (green) showing leukocytes in the different layers of the cerebellar cortex of both WT and PCD mice; nuclei are stained with DAPI (blue). **(G)** Chart showing the quantification of leukocytes per section of cerebellar vermis. **(H,I)** Graphs showing the proportional distribution of leukocytes in molecular and Purkinje cell layers **(H)** or granular layer plus the underlying white matter **(I)**; these results are complementary. Note that differences in both the quantification and distribution were detected when the degeneration of Purkinje cells is in progress (from P20 to P30). Data are expressed as dot plots, where each dot represents one individual animal and horizontal lines represent the mean of each group. *p < 0.05; **p < 0.01. GL, granular layer; PCL, Purkinje cell layer; ML, molecular layer. Scale bar: 25 μm.

As in the OB, leukocytes were found in all cerebellar layers of both WT and PCD mice (Figures 2A–F). However, in this case, the quantitative analysis determined a statistically significant increase in the number of leukocytes in PCD mice exclusively at P25 (*p* = 0.017) and P30 (*p* = 0.027), when compared with WT ones (Figure 2G). Both ages correspond to advanced neurodegenerative stages in PCD mice. This is indicative that the degree of the neurodegenerative process in each brain area is related to the extent of cell recruitment.

In addition, a striking peak in the number of leukocytes appeared at P20 in both WT and PCD mice (Figure 2G), which was statistically higher in relation to the rest of the ages of WT mice (*p* = 0.038 for P20 vs. P15, *p* = 0.002 for P20 vs. P25, *p* = 0.004 for P20 vs. P30, and *p* < 0.001 for P20 vs. P40). This is probably connected with developmental refinements occurring in the cerebellum at this age.

Given that the number of leukocytes in the cerebellum was not as limited as in the OB, we considered it appropriate to assess their distribution within the different layers of the cerebellar cortex and the underlying white matter. For this evaluation, we studied the molecular and Purkinje cell layers together (ML + PCL), where Purkinje cell dendritic arbors and somas are located, and, separately, the granular layer and the white matter (GL + WM). The percentage of leukocytes was notably higher in the ML + PCL in PCD mice regarding WT mice at P20 (*p* = 0.003), P25 (*p* = 0.002) and P30 (*p* = 0.019), i.e., throughout the cerebellar degenerative process (Figure 2H). The results concerning the percentage of leukocytes in GL + WM were the opposite (Figure 2I). All these data suggest that the loss of Purkinje cells modifies the infiltration pattern of peripheral immune cells.

### Amount, Distribution, and Characterization of Leukocytes in the Mouse Cerebellum Under Several Neuroinflammatory Scenarios

Next, we wanted to compare the effect of selective cerebellar neurodegeneration on the recruitment and distribution of leukocytes with other types of brain inflammation. Thus, we compared the following experimental paradigms: lack of inflammation (WT), inflammation associated with selective neurodegeneration (PCD), inflammation induced by LPS and inflammation induced by gamma radiation. The objective of this comparison was to ascertain whether the previously observed effects are specifically due to the degeneration of Purkinje cells. All groups were studied at P25, when the greatest difference in leukocyte infiltration and distribution between WT and PCD mice was appreciated.

**FIGURE 3:**
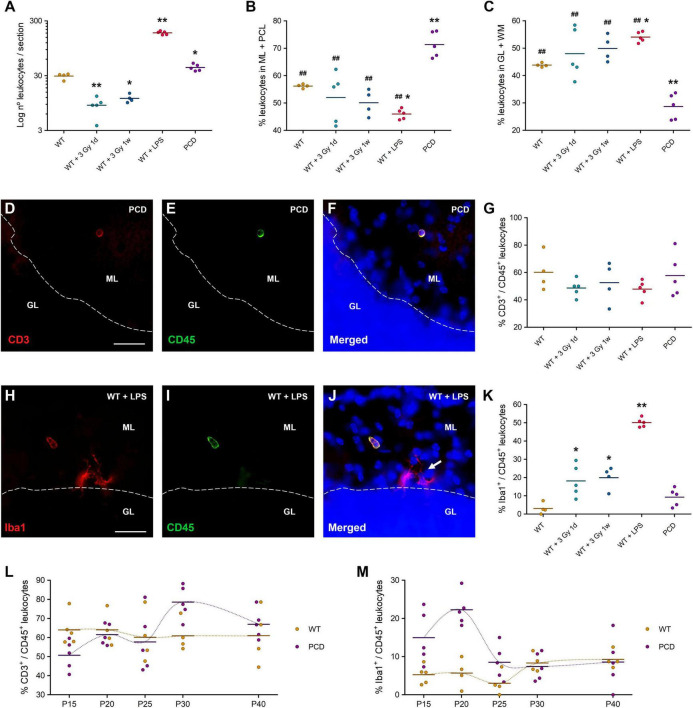
Leukocyte characterization in the cerebellum of different animal models of neuroinflammation. **(A)** Quantification of the number of leukocytes per section of cerebellar vermis. In this case a logarithmic scale was used to achieve a better visualization of the data, since the number of leukocytes in LPS-treated mice was much higher than in the other groups. **(B,C)** Distribution of leukocytes in ML + PCL **(B)** and GL + WM **(C)**; these results are complementary. Note the remarkable differential distribution of leukocytes in PCD mice. **(D–F)** Immunolabeling for CD3 (red) and CD45 (green) of a T cell in the molecular layer of a PCD mouse. **(G)** Percentage of T cells in the cerebellum. **(H–J)** Immunolabeling for Iba1 (red) and CD45 (green) of a monocyte in the molecular layer of a LPS-treated mouse. Arrow: microglial cells, which were negatively marked with CD45. **(K)** Percentage of monocytes in the cerebellum; note that radiation-induced or LPS-induced inflammation triggered monocyte infiltration, which was extremely low in both WT and PCD mice. **(L,M)** Temporal analysis of the percentage of T cells **(L)** and monocytes **(M)** in the cerebellum of WT and PCD mice; the crossing between temporal lines (dotted lines joining the mean values of each age) in both charts is indicative of the interaction between the factors analyzed. Note the fluctuations over time in PCD animals, while WT mice showed a relatively constant percentage of both T cells and monocytes throughout the whole period studied. Data are expressed as dot plots, where each dot represents one individual animal and horizontal lines represent the mean of each group. *p < 0.05 and **p < 0.01, regarding WT mice; ^##^*p* < 0.01, regarding PCD mice. Scale bars: 25 μm.

There was an evident decrease in the number of leukocytes in the cerebellum of irradiated animals with respect to the WT mice, regardless of the time elapsed from irradiation to tissue analysis, either 1 day (*p* = 0.002) or 7 days (*p* = 0.017) after treatment (Figure 3A). By contrast, leukocyte infiltration increased dramatically in LPS-treated mice as compared to the WT animals (*p* < 0.001; Figure 3A). These results point to moderate whole-body irradiation or LPS as potential agents for hampering or facilitating leukocyte infiltration into the cerebellum, respectively.

As noted above, the leukocytes in PCD mice clearly tended to localize in those layers of the cerebellar cortex mostly affected by PCD in relation to WT mice (Figures 3B,C). Interestingly, this phenomenon was not observed in LPS-treated or irradiated mice (Figures 3B,C). In fact, LPS-mediated inflammation even induced an opposite, albeit slighter, effect on the leukocyte disposition, toward the innermost layers of the cerebellum (*p* = 0.032). If we compare PCD mice with respect to the others, the differential effect on leukocyte distribution is even more evident (*p* = 0.002 for WT mice, *p* < 0.001 for irradiated and LPS-treated mice; Figures 3B,C). Representative images of the amount and distribution of leukocytes in each experimental group can be appreciated in Supplementary Figure 2. Altogether, these findings verify that the selective degeneration of Purkinje cells triggers a specific attractive effect on peripheral leukocytes.

Additionally, we wanted to characterize the leukocytes in all of these experimental groups. In particular, we studied the percentage of T cells (CD3^+^/CD45^+^ cells; Figures 3D–F) and monocytes (Iba1^+^/CD45^+^ cells; Figures 3H–J) in these scenarios, since they constitute the main peripheral immune populations that enter the brain parenchyma under neuronal damage (González and Pacheco, 2014; Solleiro-Villavicencio and Rivas-Arancibia, 2018). No statistical changes could be observed in the percentage of T cells among the experimental groups (one-way ANOVA *p*-value = 0.476; Figure 3G). Regarding the monocyte population, there was a significant increase in the percentage of monocytes in both irradiated groups in comparison with WT animals (*p* = 0.025 and *p* = 0.032 for 1 day and 7 days post-irradiation, respectively), which was even higher in LPS-treated mice (*p* < 0.001; Figure 3K). In contrast, no differences between PCD and WT animals were detected (*p* = 0.465; Figure 3K). These findings suggest that PCD does not alter the ratio of infiltrated T cells or monocytes in the cerebellar parenchyma as other less specific neuroinflammatory models do.

Although no differences in the percentage of both T cells and monocytes between the cerebellum of PCD and WT mice were identified at P25, we wanted to complete this study by analyzing possible temporal changes in both genotypes throughout the degenerative process of mutant animals. In both cases, interaction between age and genotype was detected (two-way ANOVA: *p* = 0.090 for T cells, and *p* = 0.003 for monocytes), hence no reliable further statistical analyses can be performed. Accordingly, these results are described alluding to their graphic representation (Figures 3L,M). In WT animals, the percentage of T cells and monocytes remained constant over time (Figures 3L,M). On the contrary, in PCD mice the percentage of T cells presented some fluctuations at P15 and P30 (Figure 3L). Regarding the percentage of monocytes, remarkable high cell levels in PCD mice were identified between P15 and P20 which rapidly reverted to WT levels (Figure 3M). This could well mean that initial stages of the neurodegenerative process induce a prompt and transient recruitment of monocytes into the mutant cerebellum. Altogether, these results reflect certain alterations in two of the main leukocyte types that can be found in the central nervous system, before and during the degeneration of Purkinje cells in PCD mice.

### Phenotypic Analysis of Peripheral Immune Cells

Finally, since the degeneration of Purkinje neurons exerted a special influence on the recruitment of immune cells, we decided to explore whether the phenotype of these cells is also altered during the cerebellar degeneration. To do so, the percentage of several splenic leukocyte populations was evaluated by flow cytometry, as well as the mean fluorescence intensity (MFI) of diverse myeloid and lymphoid markers. The analyzed cells were obtained from both WT and PCD spleens at P15, P20, P30 and P40. To facilitate comprehension only the significant results will be presented, which correspond to the myeloid compartment (CD11b^+^ cells). Accordingly, all the percentages were provided with respect to CD11b^+^ cells.

**FIGURE 4:**
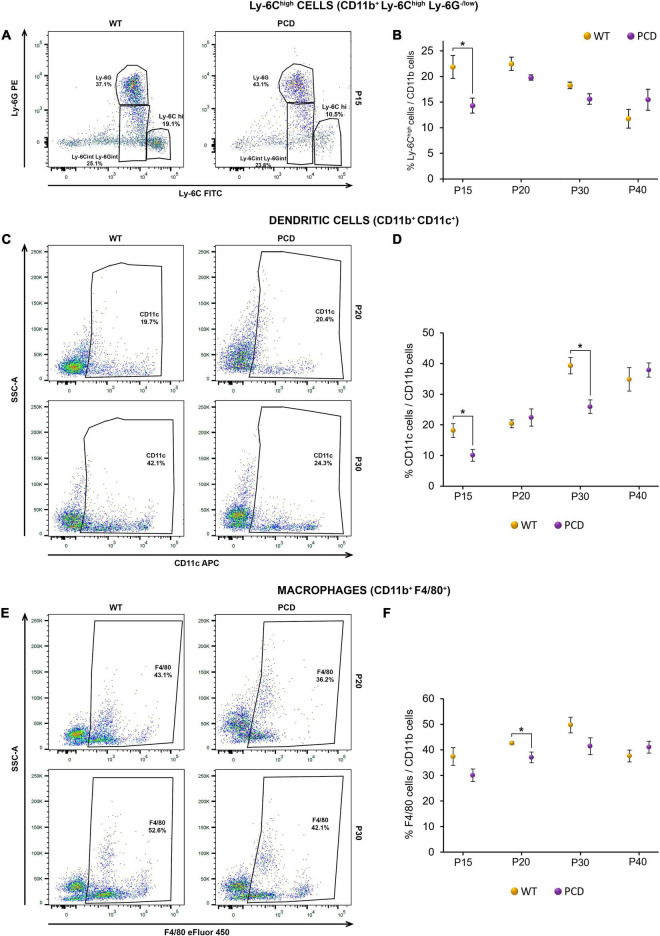
Flow cytometry analysis of the percentage of different myeloid subsets in the spleen of WT and PCD mice. **(A,C,E)** Representative dot plots showing the Ly-6C^high^ cell **(A)**, dendritic cell **(C)** and macrophage **(E)** content within the whole myeloid population after having excluded dead cells, debris and doublets. **(B,D,F)** Charts showing the percentage of the Ly-6C^high^ cells **(B)**, dendritic cells **(D)** and macrophages **(F)** within the myeloid population. Note that PCD mice presented an overall reduction in the percentage of several myeloid populations. Data are expressed as mean ± standard error of the mean. *p < 0.05.

On one hand, we found a lower percentage of several myeloid cell subsets. For instance, the percentage of inflammatory monocytes, known as Ly-6C^high^ (CD11b^+^ Ly-6G^–/low^ Ly-6C^high^), was significantly reduced in PCD mice at P15 (*p* = 0.015; Figures 4A,B), before PCD had begun. Interestingly, there were no differences at the other ages studied (Figure 4B). Furthermore, the percentage of dendritic cells (CD11b^+^ CD11c^+^) and macrophages (CD11b^+^ F4/80^+^) showed variations between the two animal groups. In the case of dendritic cells, the percentage was significantly reduced at P15 (*p* = 0.034) and P30 (*p* = 0.011; Figures 4C,D), while the percentage of macrophages presented a generalized reduction from P15 to P30, although it was only statistically significant at P20 (*p* = 0.033; Figures 4E,F).

**FIGURE 5:**
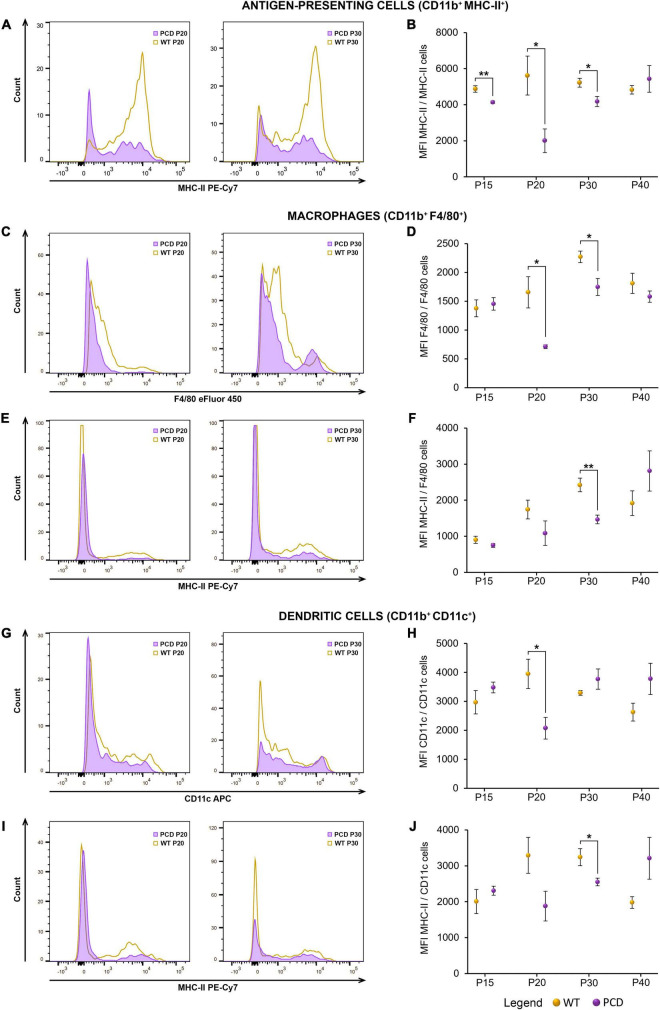
Flow cytometry analysis of the MFI of different myeloid markers in the spleen of WT and PCD mice. **(A,C,E,G,I)** Representative histograms showing the distribution of the fluorescence intensity of several myeloid markers: MHC-II within the whole antigen-presenting cell population **(A)**, F4/80 and MHC-II respect to the whole macrophage subset **(C,E)**, and CD11c and MHC-II respect to the whole dendritic cell population **(G,I)**. **(B,D,F,H,J)** Charts showing the MFI of the markers and populations described in **(A,C,E,G,I)**. Note that the myeloid cells of PCD mice exhibited a general reduction in the MFI of several myeloid markers. Data are expressed as mean ± standard error of the mean. *p < 0.05; **p < 0.01.

On the other hand, in PCD animals, several alterations in the MFI of markers related to antigen presentation and functional maturity of myeloid cells were detected. In this sense, antigen-presenting cells (CD11b^+^ MHC-II^+^) displayed a significantly reduced surface expression of MHC-II in PCD mice from P15 to P30 (*p* = 0.009 at P15, *p* = 0.048 at P20, *p* = 0.045 at P30; Figures 5A,B). Besides, the MFI of both F4/80 and MHC-II was down-regulated in macrophages (Figures 5C–F), at P20 (*p* = 0.033) and P30 (*p* = 0.042) for F4/80, and at P30 for MHC-II (*p* = 0.006). Moreover, a significant decrease of the MFI of both CD11c and MHC-II was detected in dendritic cells at P20 (*p* = 0.040) and P30 (*p* = 0.032), respectively (Figures 5G–J). Altogether, these findings reflect alterations in the peripheral myeloid compartment during the PCD that may also appear prior to the beginning of the neurodegenerative process.

Concerning the lymphoid lineage, we did not find statistically significant differences in any of the parameters studied (data not shown): the percentage of CD3^+^, CD4^+,^ and CD8^+^ cells with respect to all splenocytes, the percentage of CD4^+^ and CD8^+^ cells respecting the CD3^+^ population, the percentage of CD25^+^ and CD69^+^ (T-cell activation markers) cells respecting the CD3^+^, CD4^+^, or CD8^+^ subsets, the MFI of CD25 respecting the CD3^+^CD25^+^, CD4^+^CD25^+^, or CD8^+^CD25^+^ subsets, and the MFI of CD69 respecting the CD3^+^CD69^+^, CD4^+^CD69^+^, or CD8^+^CD69^+^ subsets.

## Discussion

The purpose of this study was to investigate how selective neurodegeneration and its associated inflammation influence peripheral immune cells. To this end, we analyzed the presence and distribution of leukocytes in the central nervous system of PCD and WT mice, as well as their phenotype at the peripheral level in the spleen. The PCD mouse was the chosen model of neurodegeneration, since it experiences the selective death of specific neuronal populations in different regions and at well-defined moments (Mullen et al., 1976; Wang and Morgan, 2007). Another point favoring the use of PCD mice, is that analogous mutations have recently been discovered in humans showing quite similar symptoms (Shashi et al., 2018; Karakaya et al., 2019; Sheffer et al., 2019). Moreover, we employed two models of general brain damage, one induced by a physical agent (gamma radiation) and the other by a biological agent (LPS), with the aim of comparing the inflammation of PCD mice (due to a selective neuronal death) with other standardized neuroinflammatory scenarios.

The selective neurodegeneration afflicting the PCD mouse cerebellum induced a specific attractive effect on leukocytes toward the cerebellar layers where dendritic arbors and somas of Purkinje cells are located, namely the molecular and Purkinje cell layers. These data agree with the distribution adopted by reactive microglial cells in the cerebellar cortex of PCD mice, which are mainly located in the Purkinje cell and molecular layers (Kyuhou et al., 2006; Baltanás et al., 2013). Furthermore, widespread inflammation or damage did not alter the cerebellar leukocyte distribution (irradiated animals) or even prompt an opposite effect (LPS-treated animals). The latter may be linked to the fact that the innermost layers are more densely populated by microglial cells (Vela et al., 1995), which constitute the main source of inflammatory mediators. Altogether, these facts further corroborate the specific attractive effect caused by the degeneration of Purkinje cells.

In parallel, our research highlights the importance of the severity of the neurodegenerative process and its associated inflammation on leukocyte recruitment. In this regard, a larger number of leukocytes was found in the PCD cerebellum, where an exacerbated reactive gliosis occurs accompanied by oligodendrocyte apoptosis (Baltanás et al., 2013). By contrast, no differences were detected between WT and PCD OBs. This may be related to the slower degenerative process and the balanced reactive gliosis occurring in this brain region, where, in addition, the death of oligodendrocytes is not provoked (Baltanás et al., 2013).

Surprisingly, at P20 we noticed an outstanding rise in the number of leukocytes in both WT and PCD cerebella. Therefore, this phenomenon does not seem to relate to the neurodegeneration itself but is probably associated with neurodevelopmental processes. Moreover, data obtained in our laboratory have identified behavioral alterations in both genotypes at a similar age (Muñoz-Castañeda et al., 2018). Indeed, around this age, a strong process of synaptic remodeling is happening in the mouse cerebellum, affecting both mossy and climbing fibers, which constitute the main inputs to the cerebellar cortex, as well as parallel fiber-Purkinje cell and interneuron-Purkinje cell synapses (van Welie et al., 2011; Kano et al., 2018). Interestingly, leukocytes have been reported to be involved in several functions during brain development (Tanabe and Yamashita, 2018). Specifically, T lymphocytes promote the establishment of inhibitory synapses, which are particularly numerous in the cerebellum (Beckinghausen and Sillitoe, 2019), through the action of INF-γ (Tanabe and Yamashita, 2018), a cytokine that prevents the formation of excitatory synapses and facilitates inhibitory synapses in GABAergic neurons (Kim et al., 2002; Filiano et al., 2016). In this sense, an attraction of T cells would be very consistent during a developmental stage.

The exposure of brain tissue to radiation triggers inflammatory reactions, characterized by a rapid glial and endothelial activation, the up-regulation of multiple pro-inflammatory cytokines and disruption of the blood-brain barrier (Lumniczky et al., 2017). Consequently, greater immune cell infiltration has been shown, specifically in areas directly exposed to the radiation beam (Burrell et al., 2012; Morganti et al., 2014; Moravan et al., 2016). We found, however, a significant decrease in the number of leukocytes in irradiated mice. These apparently contrasting results can be readily explained if we consider the differences in the way in which radiation is applied. In this study we used whole-body irradiation whereas the researchers who found an increased infiltration employed cranial irradiation. It has been proved that doses of radiation similar to ours cause immune cell apoptosis, with lymphocytes being the most sensitive population (Bogdándi et al., 2010). Consequently, we propose that the radiation induced the apoptosis of circulating cells in the irradiated mice, causing the observed decline of infiltrated leukocytes into the cerebellum. Moreover, a partial recovery of the immune cells 7 days after irradiation has been described by the same study (Bogdándi et al., 2010). Interestingly, we saw a lower reduction in the number of leukocytes 7 days than 1 day after irradiation, which means a partial recovery.

The cerebellar neurodegenerative environment of PCD mice did not seem to cause a substantial blood-brain barrier disruption, considering the light increase in the infiltrated leukocytes in comparison with LPS-treated mice. In addition, there were very few monocytes in the PCD cerebellum, at least during the advanced stages of the cerebellar degeneration. This fact supports our hypothesis, since infiltrated monocytes are only observed when the blood-brain barrier is impaired (Prinz et al., 2017), and not under physiological conditions or pathological situations where it is not affected (Ajami et al., 2007; Gomez Perdiguero et al., 2015). Unlike what happens in WT and PCD mice, in LPS-treated animals approximately half of the leukocytes were monocytes. Indeed, it has been verified that LPS not only induces the release of several pro-inflammatory cytokines in the central nervous system, such as TNF-α, IL-1β, IL-6, and CCL-2 (Qin et al., 2007; Cazareth et al., 2014; Fu et al., 2014), but also facilitates the opening of the blood-brain barrier (Banks et al., 2015; Varatharaj and Galea, 2017). The irradiated animals also exhibited a significant increase in the percentage of monocytes, which reflects an alteration of their blood-brain barrier because of the exposition to radiation. Also, it has been reported that CCL-2 is overexpressed, a chemokine which appears to be essential for monocyte infiltration after irradiation (Morganti et al., 2014; Moravan et al., 2016). Although the percentage of monocytes does not show statistically significant differences between WT and PCD mice at P25, it is slightly higher in PCD mice. This may explain the increase observed in the number of leukocytes respecting WT mice at P25. Moreover, apart from monocytes and T cells, other immune cell types could be involved in such increase, such as mast cells, neutrophiles, dendritic cells, B cells and NK cells (Ising and Heneka, 2018; Jones et al., 2019).

The analysis of the temporal infiltration of T cells and monocytes revealed that in WT animals, without neurodegenerative processes, the percentage of both cell types remains constant over time. By contrast, neuronal death in PCD mice provoked evident fluctuations in both T cells and monocytes, which seem to stabilize toward the final stages of neurodegeneration. In relation to T cells, alterations in their infiltration have been described in several models of neurodegeneration (Brochard et al., 2009; Browne et al., 2013; Evans et al., 2013; Sommer et al., 2017; Solleiro-Villavicencio and Rivas-Arancibia, 2018; Williams et al., 2020). However, the precise role of infiltrated T cells is quite heterogeneous, since their phenotypic and functional profile depends on both the type and stage of the neurodegenerative disease under consideration (González and Pacheco, 2014; Sommer et al., 2017). In addition, diverse subtypes of T cells may be implicated in the pathophysiology of these diseases. Thus, Th1 and Th17 cells appear to contribute to neuroinflammation and perpetuate neurodegenerative processes, whereas Th2 and Treg attenuate the neuroinflammatory response and promote a neuroprotective environment (González and Pacheco, 2014; Solleiro-Villavicencio and Rivas-Arancibia, 2018). Although the identification of the different subpopulations of T lymphocytes is beyond the scope of this study, it is evident that the behavior of this type of leukocytes is affected by the cerebellar neurodegeneration.

Regarding the monocytes, an increase at P15 and P20 in PCD mice was clearly noticeable as compared to the WT mice. Interestingly, a similar event was previously observed in the same animal model, but with regard to the neurodegenerative environment of the OB (Recio et al., 2011). In this study, an increase in the integration of Iba1^+^ cells derived from the bone marrow (analogous to monocytes) was observed in the OB of PCD mice at P60, when the loss of mitral cells begins, in comparison with WT mice. However, such increase disappeared at more advanced stages. Thus, although a more in-depth look into the blood-brain barrier of PCD mice is necessary, if there is any alteration, it must occur in an early and transient manner since monocytes quickly diminished at P25. In connection with these results, a low number of monocytes has been detected in the peripheral blood of ALS patients, which seems to correlate with their recruitment into the central nervous system. This phenomenon precisely becomes patent at the initial stage of the disease (Zondler et al., 2016; Baufeld et al., 2018). Such monocytic invasion could play a protective function, at least at the beginning of this pathology (Zondler et al., 2016). We cannot know if the early increase of infiltrated monocytes in PCD mice is exerting any function, but it may be linked to the peripheral variation of Ly-6C^high^ cells (see below).

Concerning the peripheral immune system, it has been proved that neurodegeneration and neuroinflammation can alter both innate and adaptive responses (Lucin and Wyss-Coray, 2009; González and Pacheco, 2014; Gupta et al., 2018; Solleiro-Villavicencio and Rivas-Arancibia, 2018; Fuzzati-Armentero et al., 2019). In this context, intriguing results were obtained in relation to the phenotypic analysis of peripheral immune cells of PCD animals. In general, a reduced expression of several immune markers was detected in specific myeloid populations of PCD mice during the degeneration of Purkinje cells or even before. Macrophages and dendritic cells were the most clearly affected populations, showing both reduced antigen presentation capability and reduced maturity, as reflected by a reduction in the MFI of F4/80, CD11c and MHC-II, the phenotypic markers of macrophages, dendritic cells and antigen-presenting cells, respectively. It would be conceivable to find a more activated immune system as a result of a peripheral inflammatory response to brain injury (Marcet et al., 2017) or neurodegeneration (Björkqvist et al., 2008; Boyko et al., 2017) but, surprisingly, we detected a worse myeloid functionality. Despite this, our results share similarities with other studies, which precisely have reported alterations in myeloid cells. Thus, monocytes derived from AD patients exhibited both impaired differentiation into macrophages and a limited phagocytic activity (Fiala et al., 2005; Jairani et al., 2019), which is the first step in the process of antigen presentation. Likewise, neutrophils isolated from AD patients displayed a diminished ability to phagocytize and destroy pathogens (Davydova et al., 2003; Le Page et al., 2017). Furthermore, dendritic cells were reduced in peripheral blood from patients with AD or PD. Interestingly, this descent was related to the severity of the symptoms (Abbott and Sotelo, 2000; Ciaramella et al., 2013, 2016). Because of this compromised innate immune response, we propose that PCD mice are highly likely to present a greater predisposition to develop infections by opportunistic pathogens, as it has been previously suggested for other neurodegenerative diseases (Le Page et al., 2017; Gupta et al., 2018). This could explain the fact that PCD mice present a higher mortality, a phenomenon that we have observed with some frequency in the animal facilities even when these animals receive special care due to their impaired motor coordination.

Despite these similarities, it is plausible to think that the immune alterations observed in PCD mice are linked to the deficit in the expression of *Ccp1*, the gene mutated in these animals, which encodes cytosolic carboxypeptidase 1 (CCP1; Berezniuk et al., 2012). CCP1 is essential for the stabilization of microtubules (Muñoz-Castañeda et al., 2018) and is synthesized in multiple tissues and cell types, including immune cells (Liu et al., 2017), which means that the disturbances observed in peripheral myeloid cells could be due to the *Ccp1* mutation. Interestingly, a recent study has proved that the deficiency of CCP1 or its homolog CCP6 promotes somatic cell reprogramming or, in other words, prevents cell differentiation (Ye et al., 2018). In addition, a previous report by the same authors revealed that *Ccp6*^–/–^ mice present underdeveloped megakaryocytes which give rise to dysfunctional platelets (Ye et al., 2014). Thus, we cannot discard that the *Ccp1* mutation is directly involved in the immune cell alterations observed in PCD mice. Although these arguments may explain these changes, our results strongly support that the neurodegenerative process is directly involved, since the alterations (both in splenic leukocytes and in the cerebellum itself) appear to be reversed at P40, when the Purkinje cell death is virtually concluded (Mullen et al., 1976; Wang and Morgan, 2007). In any case, further studies are required to accurately explain the immaturity exhibited by these myeloid cells in PCD mice.

Apart from a partial block in myeloid maturation, we found a lower percentage of Ly-6C^high^ cells in PCD mice only at P15, before the onset of the death of Purkinje cells. Precisely, at this age, ultrastructural and morphological changes begin to be evident in the Purkinje cells of PCD mice (Baltanás et al., 2011a,b; Muñoz-Castañeda et al., 2018). This may be connected with the increased number of monocytes observed in the cerebellum of PCD mice at P15 and P20, since Ly-6C^high^ cells are traditionally considered as pro-inflammatory monocytes with a high invasiveness capacity in different diseases of the central nervous system such as multiple sclerosis, ALS or stroke (King et al., 2009; Butovsky et al., 2012; Khan et al., 2016). On the other hand, Ly-6C^high^ cells have been defined as a highly plastic cell population that, depending on the clinical moment of each disease, may behave as pro-inflammatory or even as alternatively activated immunosuppressive cells with a powerful regulatory role over the adaptive immune response (Moliné-Velázquez et al., 2011; Giles et al., 2018; Melero-Jerez et al., 2019). In this sense, a decrease in the number of this regulatory myeloid cell type may be a sign of an exacerbated pro-inflammatory immune response with profound neurodegenerative consequences (Bok et al., 2018). This is a very interesting phenomenon, since the level of Ly-6C^high^ cells could be used as an early biomarker of the development of this selective neurodegeneration in PCD mice. Precisely, the concept that peripheral immune processes can function as biomarkers for neurodegenerative diseases has recently emerged in view of the growing amount of evidence supporting their involvement in the pathogenesis of these disorders (Ciaramella et al., 2016; Le Page et al., 2018). Therefore, the study of peripheral immune components could become an extremely useful tool for monitoring the progress of these diseases, as well as for measuring the therapeutic potential of different treatments (Björkqvist et al., 2009; Boyko et al., 2017) thus avoiding more complex or invasive explorations involving the central nervous system.

## Conclusion

Our findings show that the selective death of Purkinje cells induces a specific attractive effect on leukocytes toward and into the cerebellum of PCD mice. Additionally, our research highlights that the inflammatory state associated with a neurodegenerative process constitutes a key factor for cell homing and thus must be considered when applying cell therapy against these devastating disorders. In parallel, we identified peripheral immune alterations in our mouse model, which could serve as early biomarkers of its cerebellar degeneration and, in addition, may be responsible for an increased susceptibility to infections. The latter should be considered for maintaining the colonies of these animals or when subjecting them to certain surgical procedures. Finally, we hope that the data presented here will help to shed light on the behavior of peripheral immune cells in neurodegeneration and encourage the use of immune cells as a new diagnostic tool or even a therapeutic approach for neurodegenerative diseases.

## Data Availability Statement

The raw data supporting the conclusions of this article will be made available by the authors, without undue reservation.

## Ethics Statement

The animal study was reviewed and approved by the Bioethics Committee of the University of Salamanca (reference numbers: #00291 and #00344).

## Author Contributions

CP, DC, EW, and DD conceived the study and designed the experiments. CP, RL-G, EP-M, and LP-R performed the experiments. CP, CÁ-Z, JA, DC, EW, and DD interpreted the data. CP was a major contributor in writing the manuscript and organizing all the figures. All authors critically revised and approved the final manuscript.

## Conflict of Interest

The authors declare that the research was conducted in the absence of any commercial or financial relationships that could be construed as a potential conflict of interest.

## Publisher’s Note

All claims expressed in this article are solely those of the authors and do not necessarily represent those of their affiliated organizations, or those of the publisher, the editors and the reviewers. Any product that may be evaluated in this article, or claim that may be made by its manufacturer, is not guaranteed or endorsed by the publisher.

## Abbreviations

AD: Alzheimer’s disease
ALS: amyotrophic lateral sclerosis
CCP1: cytosolic carboxypeptidase 1
DAPI: 4’,6-diamidino-2-phenylindole
GL: granular layer
LPS: lipopolysaccharide
MFI: mean fluorescence intensity
ML: molecular layer
OB: olfactory bulb
PB: phosphate buffer
PBS: phosphate buffered saline
PCD: Purkinje Cell Degeneration
PCL: Purkinje cell layer
PD: Parkinson’s disease
WM: white matter
WT: wild-type.
