# Supplementary material for: The Selective Loss of Purkinje Cells Induces Specific Peripheral Immune Alterations
Source: Front Cell Neurosci. 2021 Nov 30;15:773696. doi: 10.3389/fncel.2021.773696 (PMC8671039; doi:10.3389/fncel.2021.773696)
Supplement: Supplementary file 1 [file Data_Sheet_1.docx]

Supplementary Material

**
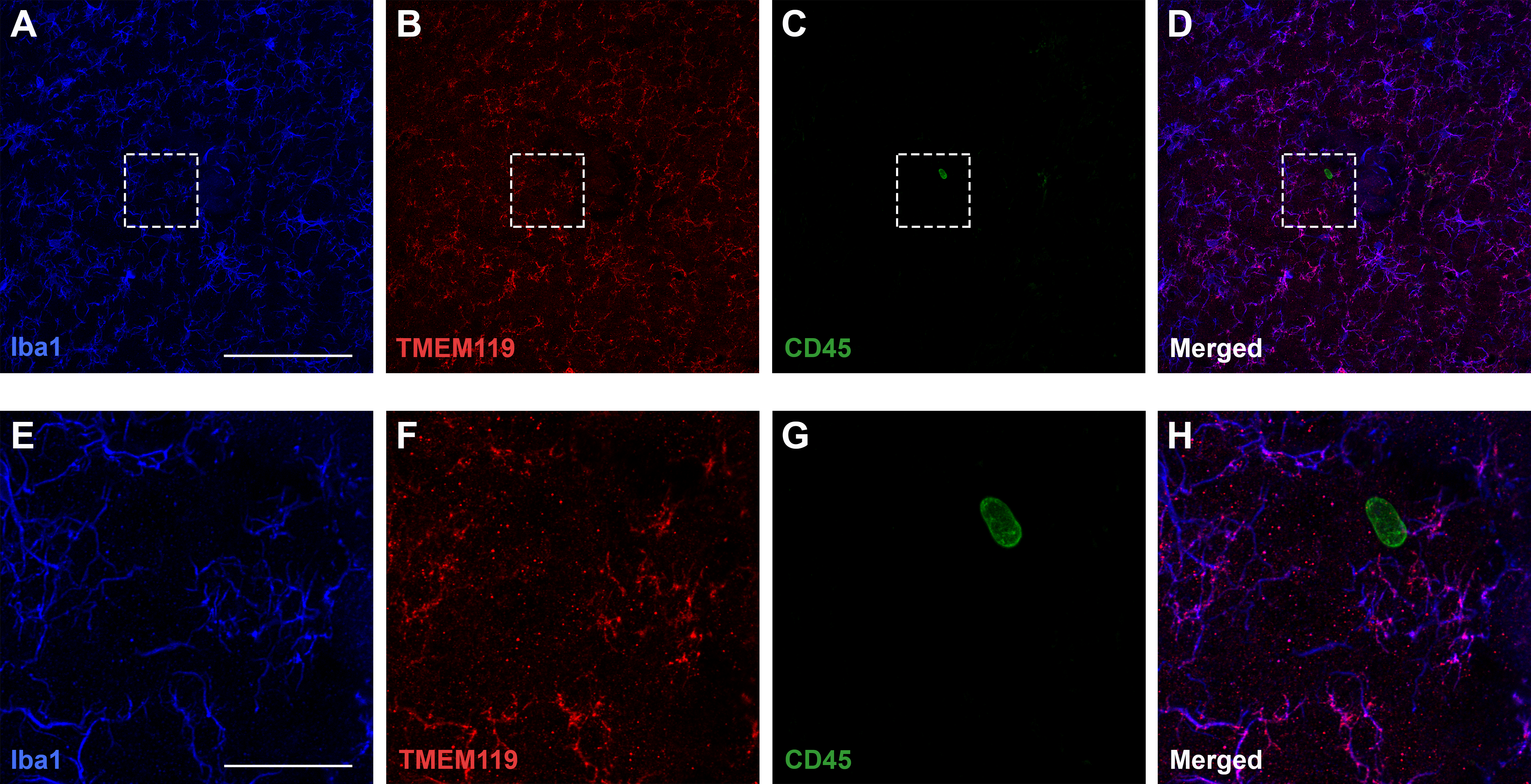
**

**Figure S1.** Characterization of the peripheral nature of a leukocyte in the mouse olfactory bulb. **(A-C)** Confocal images resulting from a triple immunohistochemistry against Iba1 (blue, **A**), TMEM119 (red, **B**) or CD45 (green; **C**). **(D)** Confocal image obtained after merging the images shown in **(A-C)**. **(E-H)** Magnification of the dotted squares depicted in **(A-D)**. Note how the rounded CD45-positive leukocyte is negatively marked for the specific microglial marker TMEM119. Scale bar: 100 μm for **(A-D)**; 20 μm for **(E-H)**.

**
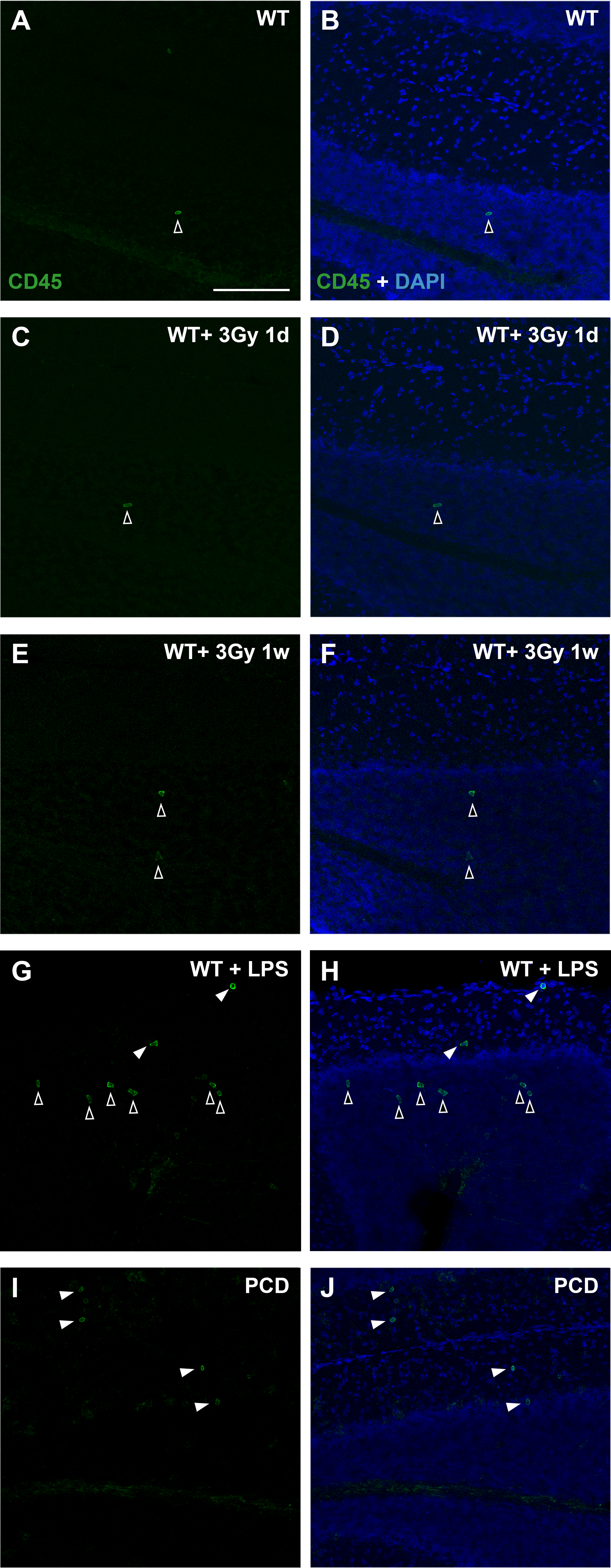
**

**Figure S2.** Representative confocal images of the infiltrated leukocytes analyzed in the cerebellum of all experimental groups: WT mice **(A,B)**, WT mice subjected to radiation 1 day **(C,D)** or 1 week before perfusion **(E,F)**, LPS-treated mice **(G,H)**, PCD mice **(I,J)**. CD45 labeling (green) is represented separately in the left column, whereas nuclei (stained with DAPI, blue) are also displayed in the right column. Filled arrowheads indicate leukocytes in the molecular or Purkinje cell layers, while hollow arrowheads indicate leukocytes in the granular layer. Note how leukocytes are more numerous in LPS-treated animals and how they are more externally located in the cerebellum of PCD mice. Scale bar: 100 μm.
